# Schistosomiasis, soil transmitted helminthiasis, and malaria co-infections among women of reproductive age in rural communities of Kwale County, coastal Kenya

**DOI:** 10.1186/s12889-022-12526-0

**Published:** 2022-01-19

**Authors:** Victor Tunje Jeza, Francis Mutuku, Lydia Kaduka, Charles Mwandawiro, Janet Masaku, Collins Okoyo, Henry Kanyi, Joyce Kamau, Zipporah Ng’ang’a, Jimmy Hussein Kihara

**Affiliations:** 1 Department of Medical Sciences, Technical University of Mombasa, Mombasa, Kenya; 2 Department of Environment and Health Sciences, Technical University of Mombasa, Mombasa, Kenya; 3 Center for Publich Health Research, Kenya Medical Research Institute, Nairobi, Kenya; 4 Eastern and Southern Africa Center for International Parasite Control, Kenya Medical Research Institute, Nairobi, Kenya; 5 Office of the Deputy Vice Chancellor, South Eastern Kenya University, Kitui, Kenya

**Keywords:** *S. Haematobium*, STH, Malaria, Co-infections, WRA, Coastal Kenya

## Abstract

**Background:**

*Schistosoma haematobium*, soil transmitted helminthes (STH), and malaria lead to a double burden in pregnancy that eventually leads to poor immunity, increased susceptibility to other infections, and poor pregnancy outcomes. Many studies have been carried out on pre-school and school aged children but very little has been done among the at risk adult population including women of reproductive age (WRA). Our current study sought to establish the risk factors and burden of co-infection with *S. haematobium*, STH, and *Plasmodium* sp. among WRA in Kwale County, Coastal Kenya.

**Methods:**

A total of 534 WRA between the ages of 15–50 were enrolled in this cross-sectional study from four villages; Bilashaka and Mwaluphamba in Matuga sub-County, and Mwachinga and Dumbule in Kinango sub-County. Socio-demographic information was collected using a pre-tested standardized questionnaire. Parasitological examination was done using urine filtration method for *Schistosoma haematobium*, Kato Katz for STH (*Ascaris lumbricoides*, Hookworm, *Trichuris trichiura*), and standard slide microscopy for *Plasmodium* sp. Statistical analyses were carried out using STATA version 15.1.

**Results:**

The overall prevalence of *S. haematobium* was 3.8% (95% CI: 2.6–5.4) while that for malaria was 4.9% (95% CI: 2.0–11.7). The prevalence of STH was 5.6% (95% CI: 2.8–11.3) with overall prevalence of 5.3% (95% CI: 2.5–10.9) for hookworm and 0.6% (95% CI: 0.2–1.9) for *T. trichiura.* The occurrence of co-infection was low and was recorded between *S. haematobium* and *P. falciparum* (0.6%), followed by *S. haematobium* and STH (0.4%).

Among pregnant women, 2.6% had co-infection with *S. haematobium* and *P. falciparum.* Only 1.3% had co-infection with *S. haematobium* and hookworm or *T. trichiura*. Among non-pregnant women, co-infection with *S. haematobium* and *P. falciparum* was 0.2%. Similarly, co-infection with *S. haematobium* and hookworm or *T. trichiura* was 0.2%.

Bed net ownership and usage among pregnant women was 87.8 and 96.6%, respectively. 66.3% of the women reported using improved water sources for drinking while 78.1% reported using improved sanitation facilities.

**Conclusion:**

The use of improved WASH activities might have contributed to the low prevalence of STHs and *S. haematobium* infections. Further, bed net ownership and usage might have resulted in the low prevalence of *Plasmodium* sp. infections observed.

**Supplementary Information:**

The online version contains supplementary material available at 10.1186/s12889-022-12526-0.

## Background

Schistosomiasis is one of the neglected tropical diseases (NTDs) caused by trematodes of the genus *Schistosoma* that infects a human host after coming in contact with water infested with snails habouring human schistosome cercariae. Three major species of the genus *Schistosoma* are major contributors to schistosomiasis in humans around the world, viz.; *Schistosoma mansoni*, *Schistosoma japonicum*, and *Schistosoma haematobium* [1]. Schistosomiasis causes morbidities and mortalities with an estimated 230 million people affected worldwide [2], making it one of the NTDs causing huge socio-economic and public health concerns. The most affected are the poor in developing countries especially in sub-Saharan Africa. Poverty is both cause and effect of the disease burden, consequently, schistosomiasis survives in poverty stricken areas, that are remote with little or no safe water and sanitation, and scarce or non-existence of health care facilities [3]. Schistosomiasis affects people across all age groups and gender. However, it affects children and women more than men and the effects of infection tend to be greater in women of reproductive age (WRA) and especially pregnant women [3, 4]. It has been shown that worms, which include *S. haematobium* and soil transmitted helminthes (STH), and malaria lead to a double burden in pregnancy that eventually leads to reduction in immunity, increased susceptibility to other infections, and poor pregnancy outcomes such as low birth weight and other developmental problems [4–6].

Approximately 40 million women of reproductive age are infected with *S. haematobium*, *S. japonicum*, and/or *S. mansoni* around the world [7, 8]. In Kenya, an estimated 6 million people are infected with schistosomiasis [9], with the majority being women and children, while another approximately 10 million people are infected with STH [10]. The prevalence of *S. haematobium* in Kwale (similar study site to the current study) among adults was 18.2% in 2010 [11] and 15.07% in 2018 among people of all age groups and gender [12]. Three major STH infections are caused by roundworms (*Ascaris lumbricoides*), whipworms (*Trichuris trichiura*), and the hookworms (*Ancylostoma duodenale* and/or *Necator americanus*) [13–15]. Generally a combination of factors have been implicated in STH infections which include poverty, lack of sanitation, and inadequate hygiene [15–17]. Consequently, combating these factors tends to reduce the infections. It has also been shown that school based deworming (SBD) reduces the burden of STH infections. However, Anderson et al [18] have shown that SBD alone may not be very effective at reducing or interrupting transmission at the community level. A number of studies have shown that SBD in combination with community based deworming (CBD) where mass drug administration (MDA) to the community is done is the key to effectively control STH infections [19, 20]. The Tuangamize Minyoo Kenya Imarisha Afya (TUMIKIA) project, a cluster randomized trial done in Kwale County to determine the effectiveness of SBD and CBD also concurs with the fact that MDA at the community level is more effective in reducing the prevalence and intensity of STH infection [20–25]. Notably, most STH infections in the study area occur in young children with low prevalence recorded in the adult population. In a study in Kwale published in 2015, Kihara et al [26] found a prevalence of 2.6% among women of reproductive age.

Malaria (*Plasmodium* sp.) infection, which is also transmitted in areas where schistosomiasis occurs, has been diagnosed together with schistosomes. Although not a NTD, malaria is a common infection in Africa and of public health concern. It affects the poor and also contributes to poverty through lost savings, lost man hours, reduction of income, expenses for preventive measures, and deaths, leading to reduction in human resource. Malaria caused an estimated 229 million clinical cases and an estimated 409,000 deaths in 2019 [27] with an estimated 90% of those deaths occuring in sub-Saharan Africa [27]. The most affected are young children who have not fully developed their protective immunity and pregnant women whose immunity to malaria is down-regulated. The prevalence of malaria was recorded as 0 % among women of reproductive age in 2015 in Kwale [26] and 6% nationally among children aged 6 months to 14 years in 2020 [28].

A significant number of studies have been done in Africa in general and Kenya in particular on schistosomiasis, STH, and malaria. The coastal region of Kenya has particularly received a fair share of these studies on schistosomiasis due to its endemicity in the region [29]. Most of the studies have however, concentrated on pre-school and school aged children [30–39] and very little has been done among the adult population including WRA. Our current study aimed at understanding the risk factors and burden of co-infection with *S. haematobium*, STH, and *Plasmodium* sp. among WRA in Kwale County, coastal region of Kenya.

## Methods

### Study design and study site

This cross-sectional study was carried out in November of 2018 in Kwale County, South Coast, Kenya. The County borders Taita Taveta County to the West, Kilifi County to the Northwest, Mombasa County to the North, the Indian Ocean to the East, and the Republic of Tanzania to the South [40]. The county is located in the Southeastern corner of Kenya, at Latitudes 3^o^ 3'’and 4^o^ 45′ South and longitudes 38^o^ 31'and 39^o^ 31′ East [40]. Kwale County is divided into 5 administrative sub-counties; Kinango, Lunga Lunga, Matuga, Msambweni, and Samburu according to the 2019 Kenya Population and Housing Census [41]. Kwale is usually hot and dry from January to March. Monsoonal heavy rains begin in mid-March and last up to June. The months of June, July, August, and September are usually cool with July being the coldest. Short rains start in October and end in early December [42]. Majority of the population practice subsistence farming of crops such as maize and cassava. A few cash crops are farmed which include coconuts, oranges, and mangoes. The area is dotted with dams and seasonal streams [26].

### Study population and sample size

**Fig. 1:**
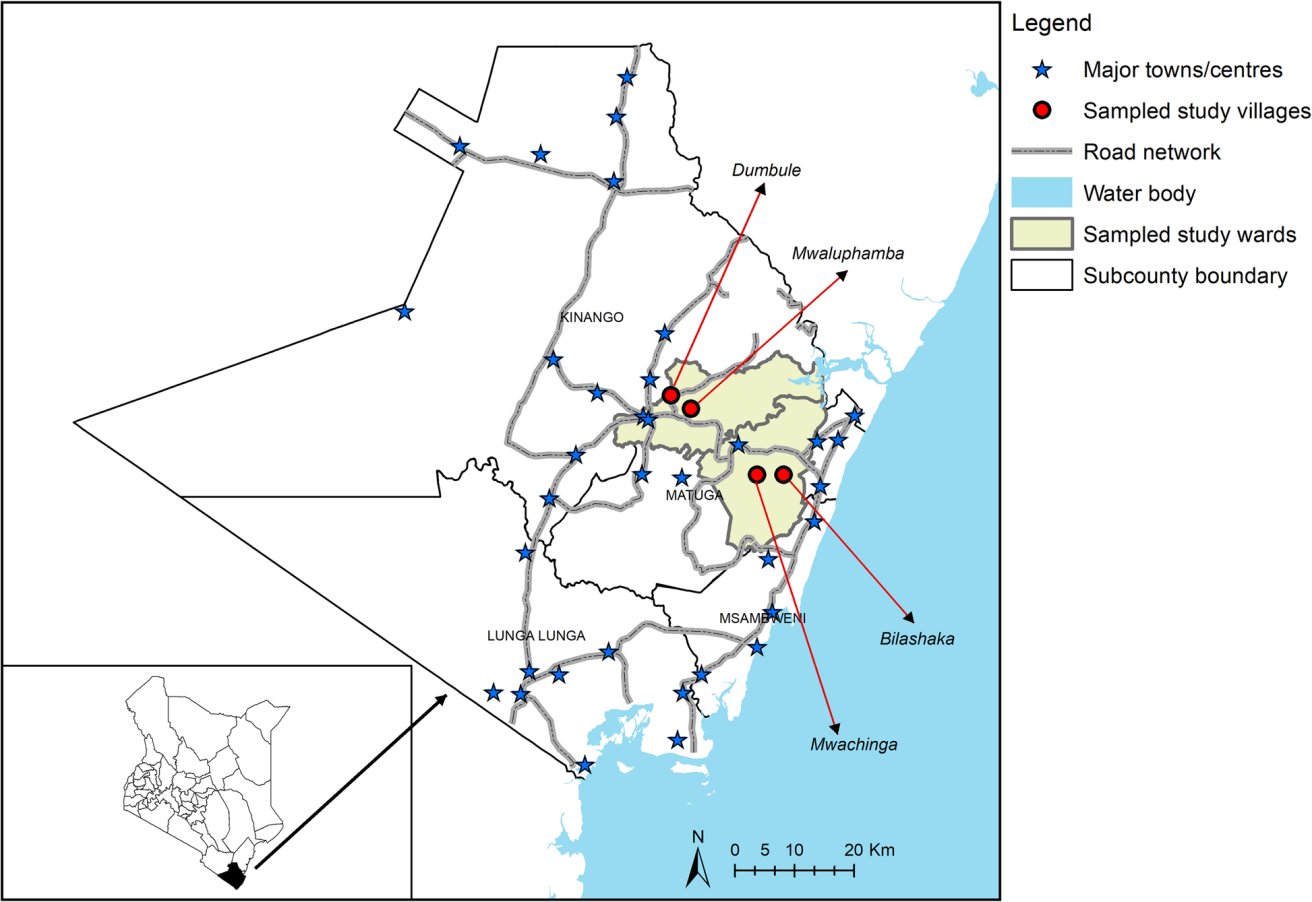
Study site: A map of the study site showing the four villages; Bilashaka, Mwaluphamba, Dumbule, and Mwachinga

According to the 2019 Kenya Population and Housing Census, Kwale County had a total population of 858,748 by 2019 [41]. The sample size of WRA was calculated using the Cochran formula [43]; *n* = *Z*^2^*pq*/*e*^2^; where Z is the score for a 5% type 1 error for a normal distribution (Z = 1.96), p is the prevalence of urogenital schistosomiasis in WRA taken as 36% [26]. This prevalence (36%) was used in this study because of the similarity of that particular study by Kihara et al [26] to our current study in terms of the infection, study population, and the study site which is one of the villages where we collected data and samples from. The proportion of the population with no infection is represented by the letter q. Using a margin of error (e) of 4.5 and 19% non-response rate, the final estimated sample size was 534 WRA. Hence, a total of 534 WRA between the ages of 15–50 were enrolled in this cross-sectional study from four randomly selected villages of Bilashaka, Mwaluphamba, Mwachinga, and Dumbule (Fig. 1). The four villages under study were in Matuga and Kinango sub-counties with a population of 192,999 and 93,789 respectively [41].

### Community mobilization and sensitization

Before commencement of the study, meetings were held with the county, sub-county, location, sub-location, village leaders, and the community to sensitize them about the study. During this sensitization period, the study participants were requested to visit the nearest health facility in the area on specific dates for sample and data collection. Two villages that were served by a health facility were randomly selected in each sub-county. The selected health facilities served as sample collection centers. They included Bilashaka and Mwaluphamba in Matuga sub-County, and Mwachinga and Dumbule in Kinango sub-County. During sample collection (stool, urine, and blood), informed consent forms were signed by all participating WRA or their guardians for those below the age of eighteen.

### Socio-demographic information

Socio-demographic data was obtained from all pregnant and non-pregnant women using a pre-tested questionnaire (Supplementary file 1). Data collection was done by trained field assistants using android based mobile phones where the questionnaires had been programmed into using Open Data Kit (ODK) software. ODK [44] incorporates in-built data quality checks to reduce data entry errors making it very attractive to use.

### Parasitological examination

*S. haematobium* infection was determined by examining fresh urine samples using nuclear pore filtration technique [45, 46]. Briefly, 10 ml of urine collected from WRA between 10 am and 2 pm was filtered in duplicate through 12.0 μm (13 mm) polycarbonate membrane filters (Sterlitech, Kent, WA, USA) mounted on urine filtration chambers. The membranes were then placed on labeled slides and examined using a microscope. Mean egg counts were calculated and expressed as egg counts per 10 ml urine, then categorized as either light (≤ 50 eggs/10 ml urine) or heavy intensity (> 50 eggs/10 ml urine) according to WHO guidelines [47].

The presence or absence of STH ova in stool was determined using Kato-Katz method. Briefly, duplicate thick smears were prepared from each stool sample using a sieve and a template calibrated to contain 41.7 mg of stool and covered with cellophane strips pre-soaked in glycerol-malachite green solution. The slides were examined for STH ova using a microscope. Egg counts were multiplied by a factor of 24 to obtain the eggs per gram of stool and categorized as light, moderate, or heavy intensity as per the WHO guidelines [47].

For malaria examination, a drop of venous blood was used to make thick and thin blood smears which were prepared and stained with 2% Giemsa for 30 min, then washed in distilled water for one minute and examined under a microscope by an experienced laboratory technologist. Thick smears were used to identify parasite densities where malaria parasites were reported against 200 white blood cells counted, while thin blood smears were used to identify the species that is specific of the malaria causing parasites observed under thick blood smears. A senior technologist examined 10% of all the blood smear slides for quality assurance and quality control. Any discrepancies were repeated and reconciled then captured electronically on laboratory reporting data forms that had been programmed onto android based smart phones using ODK [44].

### Statistical analysis

Prevalence of malaria, schistosomiasis and STH were calculated and the 95% confidence intervals (CIs) determined using binomial regression model. Average intensities were determined for schistosomiasis and STH and the associated 95% CIs estimated using negative binomial regression model. The prevalence risk ratio (RR) and the incidence rate ratio (IRR) for binomial and negative binomial regression models respectively were reported. Risk factor analysis was conducted for malaria, schistosomiasis, and STH in a two-step approach. Univariable analysis was conducted using multi-level mixed effects logistic regression model at two levels; participants nested within villages selected from sub-counties in Kwale County. In this analysis, estimates were described as odds ratios (OR). Thereafter, multivariable analysis was performed using sequential block-wise approach for each outcome of interest, where the selected covariates were included and eliminated one at a time until the most parsimonious model was obtained. The adjusted odds ratios (aOR) were obtained by mutually adjusting all minimum generated variables in a multi-level mixed effects logistic regression model at two levels. Prior to performing multivariable analysis, all covariates that met the inclusion criteria of *p* < 0.09 were investigated for collinearity using pairwise correlation. A *p*-value of ≤0.05 was considered statistically significant. All the statistical analyses were carried out using STATA version 15.1 (STATA Corporation, College Station, TX, USA).

## Results

### Socio-demographic characteristics of the study population

Overall, the data was collected from 534 WRA from the sub-Counties of Matuga (251 participants) and Kinango (283 participants). Two villages were sampled from each sub-county. The mean age of the participants was 30 years (range: 15–50 years, standard deviation (SD): 9 years). Majority of the participants were aged between 21 and 30 years, 220 (41.2%), followed by those aged 31 to 40 years, 149 (27.9%). The demographic characteristics of the study population are summarized in Table 1.

**Table 1:** Reported individual and household characteristics and the number of individuals positive for STH, *S. haematobium* and malaria infections

Factor	Number examined (%)	STH infection^**a**^			***S. haematobium***	***Malaria***
		STH combined	Hookworm	***T. trichiura***		
Overall	534 (100.0%)	30 (5.6%)	28 (5.3%)	3 (0.6%)	20 (3.8%)	26 (4.9%)
**Demographic characteristics**						
Age groups						
20 years and below	100 (18.7%)	4 (4.0%)	4 (4.0%)	0	6 (6.0%)	8 (8.1%)
21–30 years	220 (41.2%)	14 (6.4%)	14 (6.4%)	1 (0.5%)	11 (5.1%)	11 (5.0%)
31–40 years	149 (27.9%)	11 (7.4%)	10 (6.7%)	1 (0.7%)	3 (2.0%)	4 (2.7%)*
41–50 years	65 (12.2%)	1 (1.6%)	0	1 (1.6%)	0	3 (4.6%)
Level of education						
None	151 (28.3%)	12 (7.9%)	12 (7.9%)	1 (0.7%)	6 (3.9%)	6 (3.9%)
Primary	313 (59.7%)	18 (5.8%)	16 (5.1%)	2 (0.6%)	11 (3.6%)	18 (5.8%)
Secondary	60 (11.2%)	0	0	0	3 (5.0%)	2 (3.4%)
Post-secondary	10 (1.9%)	0	0	0	0	0
Average income (Ksh)						
Below 15,000	461 (86.3%)	23 (5.0%)	21 (4.6%)	3 (0.7%)	17 (3.7%)	22 (4.8%)
Between 15,000–30,000	52 (9.7%)	5 (9.6%)	5 (9.6%)	0	3 (5.8%)	2 (3.9%)
Above 30,000	21 (3.9%)	2 (9.5%)	2 (9.5%)	0	0	2 (9.5%)
Marital status						
Single/divorced/widowed	121 (22.7%)	5 (4.1%)	5 (4.1%)	0	4 (3.3%)	5 (4.2%)
Married	413 (77.3%)	25 (6.1%)	23 (5.6%)	3 (0.7%)	16 (3.9%)	21 (5.1%)
Occupation						
Farmer	195 (36.5%)	12 (6.2%)	12 (6.2%)	0	9 (4.6%)	9 (4.6%)
Business	99 (18.5%)	7 (7.1%)	7 (7.1%)	0	4 (4.0%)	6 (6.1%)
Housewife/No job	177 (33.2%)	7 (3.9%)	5 (2.8%)*	3 (1.7%)	5 (2.9%)	10 (5.7%)
Salaried worker	9 (1.7%)	0	0	0	0	0
Casual laborer	23 (4.3%)	3 (13.0%)*	3 (13.0%)*	0	2 (8.7%)	1 (4.4%)
Others	31 (5.8%)	1 (3.2%)	1 (3.2%)	0	0	0
Religion						
Christian	52 (9.7%)	4 (7.7%)	4 (7.7%)	0	3 (5.8%)	3 (5.8%)
Islam	482 (90.3%)	26 (5.4%)	24 (4.9%)	3 (0.6%)	17 (3.6%)	23 (4.8%)
**Pregnancy factors**						
Pregnancy result						
Positive	77 (14.5%)	4 (5.2%)	4 (5.2%)	0	6 (7.9%)**	9 (11.7%)***
Negative	455 (85.5%)	25 (5.5%)	23 (5.1%)	3 (0.7%)	14 (3.1%)	17 (3.7%)
Age when first pregnant						
< 18 years	127 (27.2%)	10 (7.9%)	9 (7.1%)	1 (0.8%)	4 (3.2%)	6 (4.7%)
18–25 years	321 (68.7%)	18 (5.6%)	17 (5.3%)	2 (0.6%)	12 (3.8%)	17 (5.3%)
> 25 years	19 (4.1%)	0	0	0	1 (5.3%)	1 (5.3%)
Number of pregnancies in lifetime						
One	61 (13.1%)	2 (3.3%)	2 (3.3%)	0	4 (6.6%)	7 (11.5%)
Two	67 (14.4%)	2 (2.9%)	2 (2.9%)	1 (1.5%)	3 (4.6%)	4 (5.9%)
More than two	339 (72.6%)	24 (7.1%)	22 (6.5%)	2 (0.6%)	10 (2.9%)	13 (3.8%)**
Ownership of a mosquito net						
Yes	58 (87.8%)	4 (6.9%)	4 (6.9%)	0	5 (8.8%)	7 (12.1%)
No	8 (12.1%)	0	0	0	1 (12.5%)	2 (25.0%)
Given iron						
Yes	35 (53.0%)	2 (5.7%)	2 (5.7%)	0	3 (8.8%)	4 (11.4%)
No	31 (46.9%)	2 (6.5%)	2 (6.5%)	0	3 (9.7%)	5 (16.1%)
Taken deworming tablets						
Yes	24 (36.4%)	2 (8.3%)	2 (8.3%)	0	2 (8.3%)	3 (12.5%)
No	42 (63.6%)	2 (4.8%)	2 (4.8%)	0	4 (9.8%)	6 (14.3%)
Taken antimalarial tablets						
Yes	33 (50.0%)	2 (6.1%)	2 (6.1%)	0	2 (6.1%)	5 (15.2%)
No	33 (50.0%)	2 (6.1%)	2 (6.1%)	0	4 (12.5%)	4 (12.1%)
**Individual WASH factors**						
Handwash after helping child defecate						
Always	372 (69.7%)	20 (5.4%)	19 (5.1%)	2 (0.5%)	8 (2.2%)	14 (3.8%)
Sometimes	140 (26.2%)	8 (5.7%)	7 (5.0%)	1 (0.7%)	10 (7.1%)**	10 (7.1%)
Never	22 (4.1%)	2 (9.1%)	2 (9.1%)	0	2 (9.1%)	2 (9.1%)*
Handwash before preparing food						
Always	370 (69.3%)	20 (5.4%)	20 (5.4%)	1 (0.3%)	11 (2.9%)	17 (4.6%)
Sometimes	114 (21.4%)	8 (7.0%)	6 (5.3%)	2 (1.8%)	7 (6.2%)	7 (6.2%)
Never	50 (9.4%)	2 (4.0%)	2 (4.0%)	0	2 (4.1%)	2 (4.0%)
Handwash after toilet use						
Always	471 (88.2%)	26 (5.5%)	25 (5.3%)	2 (0.4%)	18 (3.9%)	24 (5.1%)
Sometimes	48 (8.9%)	2 (4.2%)	1 (2.1%)	1 (2.1%)	2 (4.2%)	2 (4.2%)
Never	15 (2.8%)	2 (13.3%)	2 (13.3%)	0	0	0
Anal cleansing material used						
Toilet paper	7 (1.3%)	1 (14.3%)	1 (14.3%)	1 (14.3%)	1 (14.3%)	1 (14.3%)
Water	521 (97.6%)	29 (5.6%)	27 (5.2%)	2 (0.4%)	19 (3.7%)	25 (4.8%)
Leaves	6 (1.1%)	0	0	0	0	0
**Household WASH factors**						
Improved water source for drinking	354 (66.3%)	21 (5.9%)	20 (5.7%)	2 (0.6%)	14 (3.9%)	22 (6.2%)
Improved water source for household use	274 (51.3%)	17 (6.3%)	16 (5.9%)	2 (0.7%)	12 (4.4%)	19 (6.9%)*
Treatment of drinking water	92 (17.2%)	5 (5.4%)	5 (5.4%)	0	6 (6.6%)**	5 (5.4%)
Methods for water treatment						
Boiling	21 (22.8%)	3 (14.3%)	3 (14.3%)	0	2 (9.5%)	1 (4.8%)
Chlorine/Bleach	66 (71.7%)	2 (3.0%)***	2 (3.0%)***	0	4 (6.2%)	3 (4.6%)
Others	5 (5.4%)	0	0	0	0	1 (20.0%)
Improved latrine facility	417 (78.1%)	28 (6.7%)**	26 (6.3%)*	3 (0.7%)	14 (3.4%)	20 (4.8%)
Shared latrine with other households	197 (36.9%)	6 (3.1%)	5 (2.5%)	1 (0.5%)	4 (2.1%)*	12 (6.1%)
**Household assets**						
Electricity	245 (45.9%)	10 (4.1%)*	9 (3.7%)*	1 (0.4%)	6 (2.5%)	11 (4.5%)
Radio	265 (49.6%)	14 (5.3%)	12 (4.6%)	2 (0.8%)	10 (3.8%)	13 (4.8%)
Television	64 (11.9%)	4 (6.3%)	3 (4.7%)	1 (1.6%)	2 (3.1%)	2 (3.2%)
Mobile phone	481 (90.1%)	28 (5.8%)	26 (5.4%)	3 (0.6%)	17 (3.6%)	22 (4.6%)
Bank account	103 (19.3%)	4 (3.9%)	4 (3.9%)	0	5 (4.9%)	4 (3.9%)
Agricultural land	503 (94.2%)	28 (5.6%)	26 (5.2%)	3 (0.6%)	20 (4.0%)	24 (4.8%)
Cows and goats	306 (57.3%)	16 (5.3%)	16 (5.2%)	1 (0.3%)	11 (3.6%)	14 (4.6%)
Chicken and ducks	486 (91.0%)	28 (5.8%)	26 (5.4%)	3 (0.6%)	19 (3.9%)	22 (4.5%)*

^a^*Ascarislumbricoides* was not included in the analysis because no cases were recorded for this particular STH species

*indicates significance at *p* ≤ 0.05, **indicates significance at *p* ≤ 0.01, ***indicates significance at *p* ≤ 0.001

### Pregnancy

Of all the surveyed participants, 467 (87.5%) reported that they had been pregnant before, and the reported average age at first pregnancy was 19.4 years (range: 13–35 and SD: 3.2 years). The average number of past pregnancies was 4.4 (range: 1–13, SD: 2.5 pregnancies), with average live births of 3.9 (range: 1–10 and SD: 2.1 live births). Further, all the participants were tested to confirm their current pregnancy status. From the results, 77 (14.5%) were found to be pregnant (Table 1). Being pregnant was associated with increased odds of *S. haematobium* infection (OR = 2.69, *p* = 0.007) (Table 2), (aOR = 2.77, *p* = 0.015) (Table 3). Pregnancy was also associated with increased odds of malaria infection (OR = 3.40, *p* = 0.016) (Table 2) (aOR = 3.33, *p* < 0.001) (Table 4).

**Table 2:** Univariable analysis of factors associated with STH, *S. haematobium* or malaria

Factor	Number examined (%)	Univariable logistic regression [OR (95%CI); ***p***-value]			
		STH combined (***n*** = 30)	Hookworm (***n*** = 28)	***S. haematobium*** (***n*** = 20)	Malaria (***n*** = 26)
**Demographic characteristics**					
Age groups					
20 years and below	100 (18.7%)	**Reference**			
21–30 years	220 (41.2%)	1.63, *p* = 0.362	1.63, *p* = 0.362	0.84, *p* = 0.345	0.60, *p* = 0.496
31–40 years	149 (27.9%)	1.91, *p* = 0.210	1.73, *p* = 0.204	0.32, *p* = 0.172	**0.31,** ***p*** = 0.020*
41–50 years	65 (12.2%)	0.38, *p* = 0.314	–	–	0.55, *p* = 0.316
**Pregnancy factors**					
Currently pregnant	77 (14.5%)	0.94, *p* = 0.899	1.03, *p* = 0.954	**2.69,** ***p*** = 0.007*	**3.40,** ***p*** = 0.000*
Age when first pregnant					
< 18 years	127 (27.2%)	**Reference**			
18–25 years	321 (68.7%)	0.69, *p* = 0.450	0.73, *p* = 0.623	1.20, *p* = 0.824	1.13, *p* = 0.686
> 25 years	19 (4.1%)	–	–	1.71, *p* = 0.090	1.12, *p* = 0.868
Number of pregnancies					
One	61 (13.1%)	**Reference**			
Two	67 (14.4%)	0.91, *p* = 0.737	0.91, *p* = 0.737	0.69, *p* = 0.771	0.49, *p* = 0.307
More than two	339 (72.6%)	2.25, *p* = 0.106	2.05, *p* = 0.171	0.43, *p* = 0.279	**0.31,** ***p*** = 0.008*
**Bednet use and ownership**					
Net ownership	58 (87.8%)	–	–	0.67, *p* = 0.797	0.41, *p* = 0.318
Net usage					
Always	56 (96.6%)	–	–	0.08, *p* = 0.202	–
Sometimes	2 (3.5%)	**Reference**			
**Individual WASH factors**					
Handwash after helping child defecate					
Always	372 (69.7%)	**Reference**			
Sometimes	140 (26.2%)	1.06, *p* = 0.928	0.98, *p* = 0.968	**3.47,** ***p*** = 0.006*	1.96, *p* = 0.160
Never	22 (4.1%)	1.76, *p* = 0.657	1.85, *p* = 0.641	4.51, *p* = 0.106	**2.55,** ***p*** = 0.021*
Handwash before preparing food					
Always	370 (69.3%)	**Reference**			
Sometimes	114 (21.4%)	1.32, *p* = 0.605	0.97, *p* = 0.947	2.15, *p* = 0.071	1.37, *p* = 0.559
Never	50 (9.4%)	0.73, *p* = 0.757	0.73, *p* = 0.757	1.38, *p* = 0.542	0.87, *p* = 0.814
Handwash after toilet use					
Always	471 (88.2%)	**Reference**			
Sometimes	48 (8.9%)	0.74, *p* = 0.769	0.38, *p* = 0.326	1.09, *p* = 0.910	0.81, *p* = 0.629
Never	15 (2.8%)	2.63, *p* = 0.267	2.74, *p* = 0.232	–	–
**Household WASH factors**					
Improved water source for drinking	354 (66.3%)	1.20, *p* = 0.714	1.29, *p* = 0.627	1.20, *p* = 0.674	2.92, *p* = 0.231
Improved water source for household use	274 (51.3%)	1.26, *p* = 0.596	1.29, *p* = 0.596	1.45, *p* = 0.460	**2.70,** ***p*** = 0.012*
Treatment of drinking water	92 (17.2%)	0.96, *p* = 0.842	1.04, *p* = 0.813	**2.15,** ***p*** = 0.003*	1.15, *p* = 0.875
Methods for water treatment					
Boiling	21 (22.8%)	**Reference**			
Chlorine/Bleach	66 (71.7%)	**0.19,** ***p*** < 0.001*	**0.19,** ***p*** < 0.001*	0.62, *p* = 0.577	0.95, *p* = 0.905
Others	5 (5.4%)	–	–	–	5.00, *p* = 0.286
Improved latrine facility	417 (78.1%)	**4.15,** ***p*** = 0.009*	**3.83,** ***p*** = 0.021*	0.64, *p* = 0.078	0.93, *p* = 0.915
Shared latrine with other households	197 (36.9%)	0.41, *p* = 0.068	0.35, *p* = 0.071	**0.42,** ***p*** = 0.016*	1.49, *p* = 0.242

*Indicates a statistically significant association

- variable omitted because of insufficient number of observations

**Table 3:** Multivariable analysis of factors associated with *S. haematobium*

Factor	Multivariable analysis [aOR, ***p***-value] (***n*** = 20)
Currently pregnant	**2.77,** ***p*** = 0.015*
Washing hands after helping a child defecate	
Always	**Reference**
Sometimes	**4.03,** ***p*** = 0.003*
Never	5.62, *p* = 0.078
Treatment of drinking water	**3.55,** ***p*** = 0.003*
Improved latrine	**0.69,** ***p*** = 0.037*

*Indicates a statistically significant association

**Table 4:** Multivariable analysis of factors associated with malaria infection

Factor	Multivariable analysis [aOR, ***p***-value] (***n*** = 26)
Currently pregnant	**3.33,** ***p*** < 0.001*
Number of pregnancies	
One	**Reference**
Two	0.39, *p* = 0.213
More than two	0.44, *p* = 0.055
Improved water sources for household sources	**3.24,** ***p*** < 0.001*
Owning chicken and ducks	**0.37,** ***p*** = 0.028*
Washing hands after helping a child defecate	
Always	**Reference**
Sometimes	1.59, *p* = 0.298
Never	**3.23,** ***p*** < 0.001*

*Indicates a statistically significant association

### STH and *S. haematobium* prevalence and mean intensity

Overall, prevalence for any STH infection was 5.6% (95% CI: 2.8–11.3), with highest infection observed in Matuga sub-County 8.8% (95% CI: 6.3–12.3), followed by Kinango sub-County 2.8% (95% CI: 0.7–12.2). For specific STH species, the overall prevalence was 5.3% (95% CI: 2.5–10.9) for hookworm and 0.6% (95% CI: 0.2–1.9) for *T. trichiura.* The overall mean intensity was 1414 eggs (95% CI: 547–3654) and 52 eggs per gram of stool (95% CI: 17–162) for hookworm and *T. trichiura* respectively*.* For the two species, mean intensities of infection were categorized as light intensity (Table 5).

**Table 5:** Prevalence and mean intensity of STH and S. *haematobium* infections by sub-county and village

Villages	STH infection^**a**^			***S. haematobium*** (***n*** = 20)
	STH combined (***n*** = 30)	Hookworms (***n*** = 28)	***T. trichiura*** (***n*** = 3)	
***Prevalence of infection, %(95%CI)***				
**Kinango sub-County**	**2.8 (0.7–12.2)**	**2.5 (0.6–9.9)**	**0.7 (0.1–4.9)**	**4.3 (2.2–8.3)**
Dumbule village	0.7 (0.1–4.9)	0.7 (9.1–4.9)	0	2.8 (1.1–7.5)
Mwachinga village	4.9 (2.4–10.2)	4.2 (1.9–9.2)	1.4 (0.4–5.6)	5.7 (2.9–11.2)
**Matuga sub-County**	**8.8 (6.3–12.3)**	**8.4 (5.4–13.0)**	**0.4 (0.1–2.4)**	**3.2 (2.7–3.8)**
Bilashaka village	10.4 (6.1–17.8)	10.4 (6.1–17.8)	0	3.5 (1.3–9.2)
Mwaluphamba village	7.4 (4.1–13.4)	6.7 (3.5–12.5)	0.7 (0.1–5.2)	2.9 (1.1–7.7)
**Overall prevalence**	**5.6 (2.8–11.3)**	**5.3 (2.5–10.9)**	**0.6 (0.2–1.9)**	**3.8 (2.6–5.4)**
***Mean intensity of infection, epg (95%CI)***				
**Kinango sub-County**	**–**	**496 (95–2587)**	**59 (8–419)**	**39 (13–118)**
Dumbule	–	77 (1–11,467)	0	17 (3–93)
Mwachinga	–	913 (108–7699)	0	61 (16–228)
**Matuga sub-County**	**–**	**2453 (1114–5403)**	**43 (7–262)**	**91 (64–129)**
Bilashaka		0	0	108 (12–962)
Mwaluphamba		1538 (273–8666)	0	76 (9–639)
**Overall mean intensity**	**–**	**1414 (547–3654)**	**52 (17–162)**	**63 (36–112)**

^a^*Ascaris lumbricoides* was not included in the analysis because no cases were recorded for this particular STH species

The overall prevalence of *S. haematobium* was 3.8% (95% CI: 2.6–5.4) with the highest infections observed in Kinango sub-County 4.3% (95% CI: 2.2–8.3), followed by Matuga sub-County 3.2% (95% CI: 2.7–3.8) (Table 5). The overall mean intensity of infection was 63 eggs/10 ml (95% CI: 36–112), and 18/542 (3.4%) had light infections while 2/542 (0.4%) had heavy infections. The highest mean intensity was recorded in Matuga sub-County with 91 eggs/10 ml (95% CI: 64–129) where 6/259 (2.4%) had light infections while 2/259 (0.8%) had heavy infections followed by Kinango sub-County with 39 eggs/10 ml (95% CI: 13–118) where all infections were of light intensity.

Prevalence of *S. haematobium* was highest in women aged below 20 years 6.0% (95% CI: 3.5–10.2), followed by those aged between 21 and 30 years, 5.1% (95% CI: 2.9–8.9) and those between 31 and 40 years 2.0% (95% CI: 0.5–7.8) (Table 5). There were no cases of any parasites among those aged between 41 and 50 years.

Amongst the pregnant women, prevalence of *S. haematobium* was 7.9% (95% CI: 4.1–15.2) while that for non-pregnant women was 3.1% (2.0–4.7). A high prevalence of *S. haematobium* was observed among women who had one pregnancy in their lifetime 4/61 (6.6%) as compared to those who had two pregnancies 3/65 (4.6%) and more than two pregnancies 10/339 (2.9%).

### Malaria prevalence and bed net usage

The overall prevalence of malaria was 4.9% (95% CI: 2.0–11.7) (Table 6), with the highest infections occurring in Matuga sub-County at 7.9% (95% CI: 3.5–18.0), followed by Kinango sub-County at 2.1% (95% CI: 2.1–2.1). The prevalence of malaria among pregnant women was 11.7% (95% CI: 3.6–37.5) and 3.7% (95% CI: 1.7–8.2) among non-pregnant women. Further, infection with malaria was highest among the younger women aged below 20 years with a prevalence of 8.1% (95% CI: 2.1–31.5), followed by those aged 21 to 30 years at 5.0% (95% CI: 1.9–12.9) (Table 6). Bed net ownership and usage among pregnant women was high, 58/66 (87.8%) and 56/58 (96.6%), respectively, compared to those who were not pregnant. Of all participants who reported owning at least a bed net, 7/58 (12.1%) were infected with malaria, compared to the 2/8 (25.0%) who reportedly did not own at least a bed net and were infected.

**Table 6:** Prevalence of malaria infections by sub-county and village

Categories	Prevalence %(95%CI) (***n*** = 26)
**Overall**	**4.9 (2.0–11.7)**
**Kinango sub-County**	**2.1 (2.1–2.1)**
Dumbule village	2.1 (0.7–6.5)
Mwachinga village	2.1 (0.7–6.5)
**Matuga sub-County**	**7.9 (3.5–18.0)**
Bilashaka village	4.3 (1.8–10.2)
Mwaluphamba village	11.0 (6.8–17.8)
**By age group**	
20 years and below	8.1 (2.1–31.5)
21–30 years	5.0 (1.9–12.9)
31–40 years	2.7 (0.8–8.9)
41–50 years	4.6 (1.6–12.9)
**Bednet ownership**	
Yes	12.1 (2.8–51.1)
No	25.0 (16.4–38.2)
**Pregnancy result**	
Positive	11.7 (3.6–37.5)
Negative	3.7 (1.7–8.2)

### Co-infections with *S. haematobium*, STH, and malaria infections

The occurrence of co-infection was low and was recorded between *S. haematobium* and *P. falciparum* (0.6% (95% CI: 0.2–2.0)), followed by that of *S. haematobium* and STH (0.4% (95% CI: 0.1–1.1)). However, there was no co-infection between malaria and STH. Additionally, none of the participants were co-infected with all the three infections.

Among the pregnant women, 2.6% (95% CI: 0.7–10.3) had co-infection of *S. haematobium* and *P. falciparum* and only 1.3% (95% CI: 0.2–10.3) had co-infection of *S. haematobium* and hookworm or *T. trichiura*. Among the non-pregnant women, co-infection of *S. haematobium* and *P. falciparum* was 0.2% (95% CI: 0–1.5%). Similarly, co-infection of *S. haematobium* and hookworm or *T. trichiura* was 0.2% (95% CI: 0–1.5%).

### Individual and household WASH factors

Over half of the women reported use of improved water sources for drinking 354 (66.3%) with use of a public tap/stand pipe being the most common source (45.7%). In Matuga and Kinango sub-counties, use of improved water sources for drinking was reportedly high at 178/251 (70.9%) and 176/283 (62.2%) respectively. Only 92 (17.2%) of the participants reportedly treated their water to make it safer for drinking. The commonly used methods for treating drinking water were; chlorination/bleach 66/92 (71.7%) and boiling 21/92 (22.8%). Treating of drinking water using chlorine was shown to reduce the odds of STH infection (OR = 0.19, *p* = 0.001) (Table 2), (aOR = 0.27, *p* = 0.001) (data not shown).

Use of an improved sanitation facility was reported by majority of the participants 417 (78.1%) and the most common sanitation type was the traditional pit latrine 328 (61.4%). Matuga sub-County, reported the highest use of improved sanitation facilities (231/251 (92.0%)) followed by Kinango sub-County (186/283 (65.7%)). Less than half of the participants reported sharing a latrine facility with other households 197 (36.9%). Use of a shared latrine facility was highest in Matuga sub-County (95/251 (37.9%)) compared to Kinango sub-County (102/283 (36.0%)).

Handwashing was reportedly mostly done after helping a child to defecate with 372 (69.6%) of the women reporting to always wash their hands while 140 (26.2%) reported washing their hands sometimes and 22 (4.1%) reported never washing their hands. Before preparing food, 370 (69.3%) reported that they always washed their hands, 114 (21.4%) reported washing hands sometimes while 50 (9.4%) did not wash their hands. After visiting the toilet, 471 (88.2%) reported to always wash their hands, 48 (8.9%) washed their hands sometimes and 15 (2.8%) never washed their hands.

## Discussion

Overall, the prevalence for any STH infection was 5.6% with the highest infection observed in Matuga sub-County at 8.8% compared to Kinango sub-County that had a prevalence of 2.8%. It is not clear why there was a variance in prevalence between these two sub-counties since demographic characteristics and WASH factors were similar. In fact, Matuga sub-County reported the highest use of improved sanitation facilities (92.0%) compared to Kinango sub-County (65.7%). Several studies [48–50] have shown a strong association between geophagy and STH infections and the difference in prevalence observed in Matuga and Kinango sub-Counties might be attributed to possible behavior differences in this regard. However, our study did not investigate geophagy factors. The overall prevalence for hookworm and *T. trichiura* were 5.3 and 0.6% respectively. Both of the STH species reported had light intensities. Compared to previous studies [16, 26, 51, 52], this prevalence was very low. A study done in Mwaluphamba, in Kwale County in 2010 found a high prevalence of hookworm (41.7%) among adults [11]. The drastic reduction in prevalence we found may be attributed to the National School Based Deworming Programme which started in 2012 up to 2017 [53, 54] targeting school age children. It may also be attributed to the TUMIKIA project which was conducted in Kwale between 2015 and 2017 [23] where albendazole was distributed annually and bi-annually both in school based and community based MDA. However, preventive chemotherapy strategies have been shown to end up with re-infections thereby reducing the gains by such programs [54]. In addition, it has been reported in other studies that the prevalence of STH infections may remain high even after mass drug administration through school based deworming programs [16, 51, 52]. Since the Mwaluphamba study that recorded a prevalence of 41.7% of hookworm among adults was done several years ago, it is possible that sanitation, health education, behavior change including use of toilets and water sources has improved ever since and therefore this might account for the low prevalence we observed in the current study. Indeed, our study found the use of improved water sources for drinking with use of public tap/stand pipe being the most common source. Use of improved sanitation facilities was also reported with the most common sanitation type being the traditional pit latrine. This view is supported by another study which reported that the availability and use of sanitation facilities was associated with a reduction in the prevalence of soil transmitted helminthiasis [15]. However, this same study also noted that preventive chemotherapy appeared to be the main strategy to control STH infections in highly endemic areas.

Our study found a general trend of increase in STH infection with increase in age except for those aged between 41 to 50 years who recorded the lowest prevalence at 1.6%. There was no significant difference of STH prevalence among pregnant women (5.2%) compared to non-pregnant women (5.5%). However, women who had more than two pregnancies in their lifetime had a high STH prevalence (7.1%) than those who had one pregnancy. Increased odds of infection were found among those who used improved latrine facilities and casual laborers. This shows that STH infection may have resulted from water contamination rather than use or lack thereof latrines. This is demonstrated by the fact that there were reduced odds of STH infection among those who were treating drinking water with chlorine (OR = 0.19; *p* = 0.001). Taken together, the results here point to parity and WASH factors as significant determinants of STH infection status in Kinango and Matuga sub-counties. This is supported by Aranzales et al and Campbell et al who showed that parity and WASH factors, respectively, are associated with STH infections [55, 56].

The prevalence for *Schistosoma* infection among the WRA in the study area was 3.8% with the highest infections observed in Kinango sub-County at 4.3%, followed by Matuga sub-County at 3.2%. The overall observed prevalence here is lower compared to two studies done several years ago in Mwaluphamba in Kwale County; Kihara et al that showed a prevalence of 36.94% among pregnant and non-pregnant women [26] while Njenga et al which showed a prevalence of 18.2% among adults [11]. A number of other studies elsewhere [45, 52, 57–59] have also reported high prevalence of *S. haematobium* infections. *S. haematobium* infection rates have also been shown to be influenced by weather patterns, and local and focal clustering patterns [60] and this may partially explain the relatively low rates observed. Further, the socio-demographic factors observed might have led to a change of the study area from high risk to a low risk site. This speculation is supported by a study done in Tanzania which found a low prevalence of 4% among women of reproductive age in low risk sites compared to 53% in high risk sites [61].

In the present study, younger women were more infected than older women and as age increased, *S. haematobium* infection rate reduced. Acquired protective immunity among older women might account for this difference. It could also be possible that younger women are doing most of the chores related to water contact such as washing clothes and fetching water and therefore are more exposed to the infection. Pregnancy was associated with increased odds of *S. haematobium* infection. A high prevalence of *S. haematobium* was observed among women who had one pregnancy in their lifetime (6.6%) compared to those who had two pregnancies (2.9%). This is different from another study that found no significant difference in the prevalence of urogenital schistosomiasis among pregnant and non-pregnant women in Kwale [26]. Our findings are, however, similar to a study done in Nigeria which showed that urogenital schistosomiasis among pregnant women was high at a prevalence of 20.8% with younger women and those pregnant at the greatest risk [62]. Similarly, the average prevalence of schistosomiasis in pregnant women in Anambra state, Nigeria, was reported to be high at 23.8% with younger (16 to 20 years old) pregnant women reported to have a higher prevalence of schistosomiasis than older pregnant women [63]. Improved usage of latrine facilities was associated with reduced odds of getting *S. haematobium* infection. Taken together, our results show that age, pregnancy, WASH factors, and poverty are risk factors contributing to *S. haematobium* infections among WRA in the current study which is in line with previous studies [55, 56, 62, 63].

The overall prevalence of malaria was 4.9% with the highest infections occurring in Matuga sub-County at 7.9% followed by Kinango sub-County at 2.1%. Our results are similar to those by Njenga *et. al*. that found a malaria prevalence of 5.6% in Kwale [11]. The prevalence of malaria among pregnant and non-pregnant women was 11.7 and 3.7% respectively. The odds of getting malaria were statistically significantly higher for women who were at the time pregnant than those who were not pregnant. We speculate that this could be as a result of the differences in immune status between the two groups of women. This is supported by several studies as reviewed by Rogerson et al. [64]. More recent data supports this and has shown that antibody responses to pregnancy specific pRBCs and VAR2CSA antigens are not correlated with protection against malaria but rather markers of infection [65]. Additional findings in our study are that those who used improved water sources for drinking had increased odds of getting malaria. This is in contrast to another study that showed that improved water and sanitation conditions were associated with a decreased risk of malaria infection [66]. It is not clear why there was this association in our study. Further studies may be needed to explain this. Interestingly, owning chicken and ducks was statistically significantly associated with reduced odds of malaria infection. It has been shown that chicken volatile compounds act as medium to long range repellents of malaria causing *Anopheles arabiensis* thereby disrupting their host seeking behavior [67]. This could partially explain the observation that owning chicken and ducks reduced the odds of malaria infection. However, more work is needed to ascertain this. Those who had more than two pregnancies in their lifetime were less likely to have malaria (OR = 0.31; *p* value = 0.008) unlike those with a single pregnancy. The prevalence of malaria was highest among younger women aged below 20 years (8.1%) followed by those aged between 21 and 30 years at 5.0%. This may be due to acquisition of protective immunity among the older population.

The occurrence of low co-infection rate between *S. haematobium* and *P. falciparum* (0.6%) and that between *S. haematobium* and STH (0.4%), could be attributed to the continuous school based deworming that has been taking place in the area for the past 7 years and the national insecticide treated bed net distribution and use by pregnant women for more than a decade. The low prevalence of co-infections found in this study compared to single infections is a trend that is supported by several studies. For example, a study done in Kingwede, Kwale County found that co-infection prevalence was lower than single infections and that children had 9.3 times the odds of co-infection compared to adults [68]. In another study by Malhotra et al [52], the prevalence of single infections was high compared to co-infection among mothers during antenatal clinic visits at Msambweni District Hospital. Similarly, a low prevalence of polyparasitism was recorded among pregnant women in Bogota, Colombia compared to single intestinal parasitism infections [56]. However, the trend was found to be different among children in a study done in Zambia which found a high prevalence of polyparasitism compared to single infections of malaria, hookworm, and *S. haematobium* [34].

There were no malaria co-infections with STH or with all the three parasitic infections in our study. This is contrary to a study by Florey et al which showed that the odds of *S. haematobium* infection increased as *Plasmodium* sp. infection intensity increased [68]. In another study done in Kwale, Njenga et al reported much higher prevalence of co-infections of *S. haematobium* with hookworm at 45.8% and that of *S. haematobium* with *T. trichiura* at 6.7% [11]. Our study found that among those who were pregnant, 2.6% had co-infections with *S. haematobium* and malaria, while 1.3% had co-infections with *S. haematobium* and STH. Among non-pregnant women, co-infection with *S. haematobium* and malaria was 0.2% which was the same with co-infections of *S. haematobium* and STH. These results suggest that pregnant women are more susceptible to co-infections than non-pregnant women. It has long been believed that during pregnancy, the mothers’ immunity is down regulated [64], which is supported by new data [65], and this may account for the disparity in prevalence among the pregnant and non-pregnant women.

This study had some limitation; firstly, the sensitivity of Kato Katz method of STH diagnosis may not pick low prevalence and intensity in an endemic area. A much more sensitive method [69] is thus recommended for future studies on prevalence and intensity of schistosomiasis and STH in the area under study. Secondly, we used a single day urine examination which may have led to missing some positive cases of *S. haematobium* [70] given the low prevalence and intensity observed in the study area. Further, given the low prevalence of parasitic infections in Matuga and Kinango sub-Counties, we needed a much larger sample size to make a more reasonable comparison of the different results; however, we were limited by the resources available for the study.

## Conclusion

Our results generally show a low prevalence of STHs, *S. haematobium*, and malaria in Matuga and Kinango sub-counties with the common infection among the WRA that participated in this study being STH followed by malaria and then schistosomiasis. Light intensities were recorded for both STHs and *S. haematobium*. Cases of co-infections were low, and occurred between schistosomiasis and malaria or STH. There were no co-infections recorded between malaria and STH. Regular school and community based treatment programs might have played a major role in reducing the STH and *S. haematobium* prevalence. Pregnant women were found to be disproportionately burdened by all the infections compared to non-pregnant women thereby supporting the fact that pregnancy is a significant risk factor for these infections in the study area. Overall, high cases of all the three infections were observed in Matuga sub-County compared to Kinango sub-County. In conclusion, our results show a general use of improved WASH factors in the study area that might have led to the low infection rates observed. Further, bed net ownership and usage were high and could have contributed to the low prevalence of malaria infections.

## Supplementary Information

**Additional file 1.**

**Additional file 2.**

## Data Availability

The datasets used and/or analyzed during the current study are available from the corresponding author on reasonable request.

## Abbreviations

*S. haematobium*: *Schistosoma haematobium*
NTDs: Neglected Tropical Diseases
STH: Soil Transmitted Helminthes
*T. trichiura*: *Trichuris trichiura*
WRA: Women of Reproductive Age
*S. mansoni*: *Schistosoma mansoni*
*S. japonicum*: *Schistosoma japonicum*
OR: Odds Ratio
aOR: Adjusted Odds Ratio
WHO: World Health Organization
HIV-1: Human Immunodeficiency Virus strain 1
WASH: Water, Sanitation, and Hygiene
SBD: School Based Deworming
CBD: Community Based Deworming
MDA: Mass Drug Administration
TUMIKIA: Tuangamize Minyoo Kenya Imarisha Afya “together we eradicate worms in Kenya for a better health”
