# Supplementary material for: Schistosomiasis, soil transmitted helminthiasis, and malaria co-infections among women of reproductive age in rural communities of Kwale County, coastal Kenya
Source: BMC Public Health. 2022 Jan 19;22:136. doi: 10.1186/s12889-022-12526-0 (PMC8772099; doi:10.1186/s12889-022-12526-0)
Supplement: Supplementary file 1 — Additional file 1. [file 12889_2022_12526_MOESM1_ESM.docx]

**Questionnaire on the work entitled: Schistosomiasis, Soil Transmitted Helminthiasis, and Malaria Co-Infections among Women of Reproductive Age in Rural Communities of Kwale County, Coastal Kenya**

**Socio-Demographic Characteristics**

**ID Code:……………………… Sub-county…………………...**

**Village………………………… County……………………….**

**Location……………………….**

1. Sex Male ( ) Female ( )

2. Age in Years _____________________

3. Marital Status

Single

Currently Married (Tick) Polygamous ( ) Monogamous ( )

Divorced

Widow/ widower

4. How old were you when you got married for the first time?

5. Level of Education (Tick)

Never attended school

Did not complete primary school

Completed primary school but did not complete secondary school

Completed secondary school

Further studies after secondary school

Others, specify___________________________

6. Religion (Tick)

Christian

Islam

Non-practicing

Others, specify__________________________

**Socio-Economic Characteristics**

7. Main occupation (Tick)

Peasant farmer

Small business (kiosk, kibanda)

Big business (shop)

Housewife

Salaried worker (teacher, police, chief)

Casual laborer

Others, specify________________________

8. What is the average monthly income for entire household?

**Water, Sanitation, and Hygiene (WASH)**

9. What is the main source of drinking water for members of your household?

10. What is the main source of water used by your household for other purposes such as cooking and handwashing?

11. Do you pay or barter for water?

12. How long does it take to go there, get water and come back in one trip?

13. Who is the main person in the household that goes to fetch water from the source?

14. Do you treat your water in any way to make it safe for drinking?

15. Do you wash your hands after helping your child defecate?

16. Do you wash your hands before preparing food?

17. Do you wash your hands after using the toilet?

18. How do you clean yourself after using the toilet (defecating)?

19. What kind of toilet facility do members of your household use?

20. Do you share this toilet facility with other households?

**Asset ownership**

21. Does your household own the following?

Electricity

Radio

Television

Mobile phone

Bank Account

Agricultural land

Cows/Goats

Chickens/Ducks

**Pregnancy outcomes**

22. Have you ever been pregnant before?

23. How old were you when you first became pregnant?

24. How many pregnancies have you had in your lifetime?

25. How many live births have you had in your lifetime?

26. Are you currently pregnant?
