# Supplementary material for: Schistosomiasis, soil transmitted helminthiasis, and malaria co-infections among women of reproductive age in rural communities of Kwale County, coastal Kenya
Source: BMC Public Health. 2022 Jan 19;22:136. doi: 10.1186/s12889-022-12526-0 (PMC8772099; doi:10.1186/s12889-022-12526-0)
Supplement: Supplementary file 2 — Additional file 2. [file 12889_2022_12526_MOESM2_ESM.docx]

**Supplementary file 2: Univariable analysis of additional factors associated with STH, *S. haematobium* or malaria**

| **Factor** | **Number examined**  **(%)** | **Univariable logistic regression [OR (95%CI); p-value]** | | | |
| --- | --- | --- | --- | --- | --- |
|  |  | **STH combined**  **(n=30)** | **Hookworm**  **(n=28)** | ***S. haematobium***  **(n=20)** | **Malaria**  **(n=26)** |
| **Demographic characteristics** |  |  |  |  |  |
| Level of education |  |  |  |  |  |
| None | 151(28.3%) | **Reference** |  |  |  |
| Primary | 313(59.7%) | 0.71, p=0.295 | 0.63, p=0.255 | 0.89, p=0.713 | 1.47, p=0.345 |
| Secondary | 60(11.2%) | - | - | 1.27, p=0.688 | 0.85, p=0.893 |
| Post-secondary | 10(1.9%) | - | - | - | - |
| Average income (Ksh) |  |  |  |  |  |
| Below 15000 | 461(86.3%) | 0.50, p=0.219 | 0.45, p=0.184 | 0.63, p=0.638 | 0.48, p=0.495 |
| Between 15000-30000 | 52(9.7%) | 1.01, p=0.971 | 1.01, p=0.971 | - | 0.38, p=0.485 |
| Above 30000 | 21(3.9%) | **Reference** |  |  |  |
| Marital status |  |  |  |  |  |
| Single/divorced/widowed | 121(22.7%) | **Reference** |  |  |  |
| Married | 413(77.3%) | 1.49, p=0.517 | 1.37, p=0.586 | 1.19, p=0.799 | 1.23, p=0.829 |
| Occupation |  |  |  |  |  |
| Farmer | 195(36.5%) | **Reference** |  |  |  |
| Business | 99(18.5%) | 1.17, p=0.846 | 1.17, p=0.846 | 0.87, p=0.741 | 1.33, p=0.468 |
| Housewife/No job | 177(33.2%) | 0.63, p=0.208 | **0.44, p=0.016*** | 0.60, p=0.367 | 1.24, p=0.586 |
| Salaried worker | 9(1.7%) | - | - | - | - |
| Casual laborer | 23(4.3%) | **2.29, p=0.038*** | **2.29, p=0.038*** | 1.96, p=0.056 | 0.94, p=0.970 |
| Others | 31(5.8%) | 0.51, p=0.244 | 0.51, p=0.244 | - | - |
| Religion |  |  |  |  |  |
| Christian | 52(9.7%) | **Reference** |  |  |  |
| Islam | 482(90.3%) | 0.69, p=0.569 | 0.63, p=0.497 | 0.60, p=0.482 | 0.82, p=0.244 |
| Given iron | 35(53.0%) | 1.14, p=0.921 | 1.14, p=0.921 | 0.90, p=0.686 | 0.67, p=0.320 |
| Taken deworming tablets | 24(36.4%) | 1.82, p=0.601 | 1.82, p=0.601 | 0.84, p=0.756 | 0.86, p=0.685 |
| Taken antimalarial tablets | 33(50.0%) | 1.00, p=1.000 | 1.00, p=1.000 | 0.45, p=0.178 | 1.29, p=0.597 |
| **Individual WASH factors** |  |  |  |  |  |
| Anal cleansing material used |  |  |  |  |  |
| Toilet paper | 7(1.3%) | **Reference** |  |  |  |
| Water | 521(97.6%) | 0.35, p=0.481 | 0.33, p=0.460 | 0.23, p=0.238 | 0.30, p=0.103 |
| Leaves | 6(1.1%) | - | - | - | - |
| **Household assets** |  |  |  |  |  |
| Electricity | 245(45.9%) | **0.57, p=0.030*** | **0.54, p=0.015*** | 0.49, p=0.247 | 0.86, p=0.591 |
| Radio | 265(49.6%) | 0.89, p=0.727 | 0.75, p=0.444 | 1.01, p=0.973 | 1.02, p=0.934 |
| Television | 64(11.9%) | 1.14, p=0.798 | 0.87, p=0.856 | 0.80, p=0.406 | 0.61, p=0.539 |
| Mobile phone | 481(90.1%) | 1.58, p=0.557 | 1.46, p=0.643 | 0.61, p=0.362 | 0.59, p=0.206 |
| Bank account | 103(19.3%) | 0.64, p=0.507 | 0.69, p=0.604 | 1.42, p=0.353 | 0.76, p=0.478 |
| Agricultural land | 503(94.2%) | 0.86, p=0.754 | 0.79, p=0.610 | - | 0.73, p=0.747 |
| Cows and goats | 306(57.3%) | 0.84, p=0.675 | 0.99, p=0.983 | 0.91, p=0.922 | 0.87, p=0.165 |
| Chicken and ducks | 486(91.0%) | 1.41, p=0.637 | 1.30, p=0.700 | 1.92, p=0.499 | **0.52, p=0.017*** |
| *Indicates a statistically significant association  - variable omitted because of insufficient number of observations | | | | | |
